# Increased Insulin/Insulin Growth Factor Signaling Advances the Onset of Metamorphosis in Drosophila

**DOI:** 10.1371/journal.pone.0005072

**Published:** 2009-04-07

**Authors:** Magdalena A. Walkiewicz, Michael Stern

**Affiliations:** Department of Biochemistry and Cell Biology, Rice University, Houston, Texas, United States of America; UT MD Anderson Cancer Center, United States of America

## Abstract

Mechanisms by which attainment of specific body sizes trigger developmental transitions to adulthood (e.g. puberty or metamorphosis) are incompletely understood. In Drosophila, metamorphosis is triggered by ecdysone synthesis from the prothoracic gland (PG), whereas growth rate is increased by insulin/insulin growth factor signalling (IIS). Transgene-induced activation of PI3K, the major effector of IIS, within the PG advances the onset of metamorphosis via precocious ecdysone synthesis, raising the possibility that IIS triggers metamorphosis via PI3K activation in the PG. Here we show that blocking the protein kinase A (PKA) pathway in the insulin producing cells (IPCs) increases IIS. This increased IIS increases larval growth rate and also advances the onset of metamorphosis, which is accompanied by precocious ecdysone synthesis and increased transcription of at least one ecdysone biosynthetic gene. Our observations suggest that IIS is regulated by PKA pathway activity in the IPCs. In addition, taken together with previous findings, our observations are consistent with the possibility that, in Drosophila, attainment of a specific body size triggers metamorphosis via the IIS–mediated activation of PI3K and hence ecdysone synthesis in the PG.

## Introduction

In organisms such as Drosophila and mammals, growth occurs during an early juvenile phase, but at a certain age (corresponding to metamorphosis and puberty, respectively), organisms transition to non-growing, sexually mature adults. How do organisms sense when they are large enough to undertake the transition to adulthood? In humans of the western world, the age at puberty has decreased over the past 150 years, a phenomenon attributed to improved nutrition and hence increased growth rate, whereas Drosophila larvae reared on poor nutrients grow slowly and exhibit developmental delays. Thus, the timing of onset to adulthood is not fixed, but rather is affected by growth rate, suggesting that the hormones that trigger growth interact with the hormones that trigger developmental transitions. How these hormones might interact at the molecular level remains incompletely understood.

In Drosophila larvae, metamorphosis is triggered by the steroid hormone ecdysone [1], which is synthesized in the prothoracic gland (PG), whereas larval growth rate is increased by insulin-like peptides (dilps) released from the insulin producing cells (IPCs) of the larval brain [2]. An effect of insulin/insulin growth factor signalling (IIS) on the timing of metamorphosis was suggested by two observations. First, genetic ablation of the IPCs, or a partial loss of function mutation in the single insulin receptor *InR*, each decreased IIS and growth rate, and also delayed metamorphosis [2], [3], suggesting that metamorphosis is activated by an IIS-responsive process. However, neither this process nor this IIS-responsive tissue was identified, and it was not clear from these experiments to what extent IIS was rate limiting for developmental progression. Second, transgene-induced PI3K activation in the PG triggered a precocious metamorphosis as a consequence of increased transcription of at least two ecdysone biosynthetic genes and hence precocious ecdysone synthesis [4]–[6]. Given that PI3K is a major target of insulin action, it was suggested specifically [6] that IIS regulates the timing of developmental transitions via PI3K activity, and hence ecdysone synthesis, in the PG. However, it was not clear to what extent the effects of transgene-induced alterations in PI3K would mirror the response of a genetically wildtype PG to altered IIS.

To address these issues, we increased IIS by transgene manipulation within the IPCs and tested the effects of this increased IIS on growth rate and the timing of metamorphosis. We found that increased IIS significantly increased growth rate but had only a modest effect on final body size. In contrast, onset of metamorphosis was sharply advanced: larvae with increased IIS underwent metamorphosis about 36 hours earlier than wildtype, and this precocious development was accompanied by precocious synthesis of ecdysone and increased transcription of at least one ecdysone biosynthetic gene. We conclude that IIS is rate limiting for metamorphosis, and advances the onset of metamorphosis even in the absence of changes to other signalling systems. Such an effect of increased IIS on the timing of metamorphosis has not been previously reported. In addition, combined with previous work demonstrating that PI3K activity in the PG is also rate limiting for metamorphosis [4]–[6], our results are consistent with the possibility that IIS accelerates development by directly activating PI3K, and hence inducing ecdysone synthesis, in the PG. Finally we conclude that under conditions of extremely rapid growth, Drosophila larvae choose extremely rapid development over formation of larger flies.

## Results

### Inhibiting the PKA pathway in the insulin producing cells increases insulin signalling

**Figure 1:**
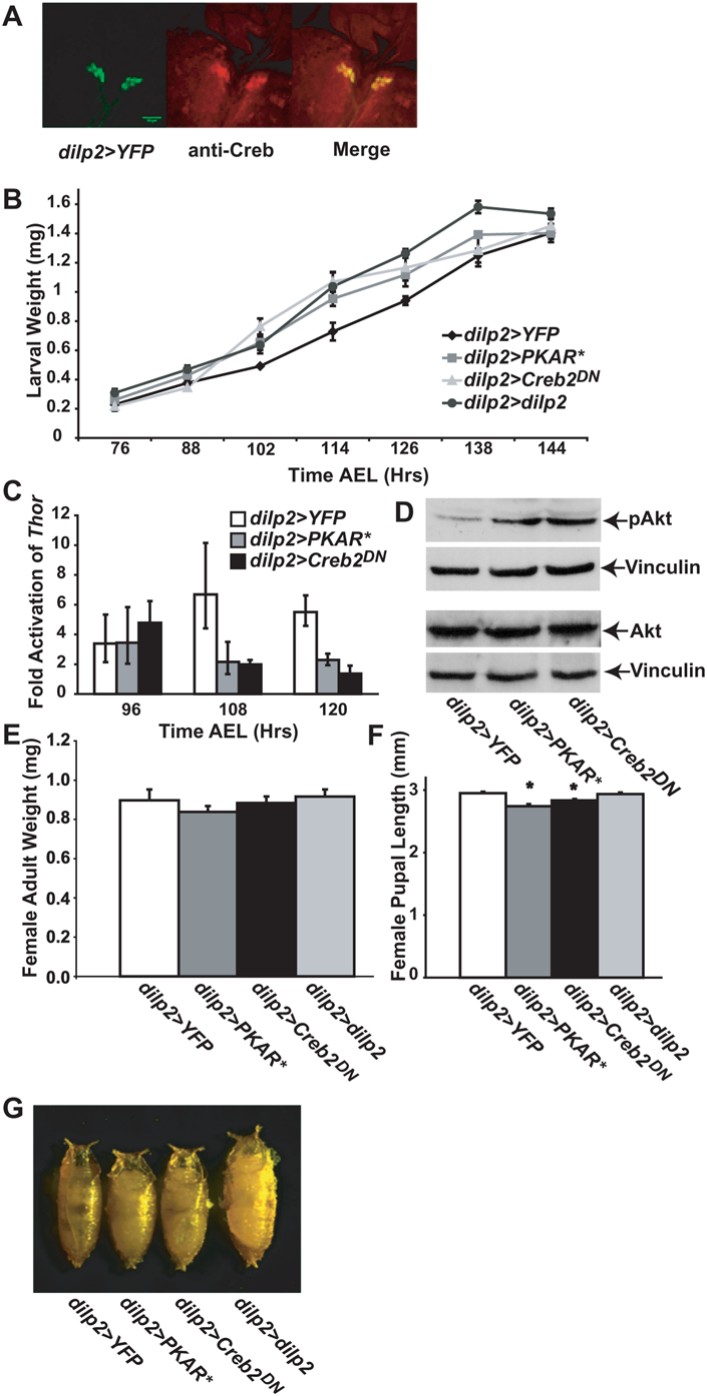
Inhibition of the PKA pathway in the IPCs increases IIS. (A) Brain lobes and ring gland from *dilp2>YFP* wandering third instar larva, showing enrichment of anti-Creb immunoreactivity in the IPCs. Red: anti-Creb, green: YFP. The scale bar is 35 µm. (B) Mean larval weight (y-axis) of the indicated genotypes (x-axis) from 76 to 144 hours after egg laying (AEL). Error bars represent SEMs. At least 3 independent biological samples of each genotype, each containing at least 3 larvae, were measured. (C) Mean *Thor* transcript levels (y-axis) were determined from two biological samples collected from each genotype (x-axis) and measured in triplicate at the indicated time AEL. Data were obtained by quantitative RT-PCR with the relative 2^−ΔΔCt^ method using the Thor and RPII140 expression assay (Applied Biosystems). Error bars represent SEMs. (D) Western blots from protein extracts obtained from developmentally staged larvae (108 hours AEL) of the genotypes indicated below the gels, using the antibody indicated to the right of each gel. The top two panels and bottom two panels represent blots made from separate gels. (E) Female adult weight (y-axis) of the indicated genotypes (X-axis). At least six biological samples each containing at least five flies were measured. Means+/−SEMs are indicated. (F) Length (y-axis) of >10 female pupae of the indicated genotypes (X-axis). Means+/−SEMs are indicated. (G) Photographs of female pupae of the indicated genotypes.

To evaluate the effects of altered IIS on growth rate and the timing of metamorphosis, we began by altering activity of genes of the protein kinase A (PKA) pathway within the insulin producing cells (IPCs). We had two reasons for hypothesizing that altered PKA pathway activity within the IPCs might alter IIS. First, PKA and its downstream transcription factor Creb activate transcription of the mammalian insulin gene [7], [8] and insulin-receptor substrate 2 [8]. Second, a dwarf body size phenotype, indicative of altered IIS, is observed in Drosophila *Creb* and *PKA* mutants [9]–[11]. Our observation that immunoreactivity to Creb is enriched in the IPCs (Figure 1A) supports the possibility of a role for PKA and Creb in IIS.

To test this possibility, we inhibited PKA signalling specifically in the IPCs by use of the *Gal4/UAS* system [12]. In particular, we used the *dilp2-Gal4* driver, which expresses specifically in the IPCs [2], [13], to induce expression of the dominant-negative PKAR* [10], which encodes a PKA regulatory subunit that fails to bind cAMP and thus constitutively inhibits PKA activity, and *Creb2^DN^* transgene [14], which encodes the b-zip dimerization domain and blocks the ability of wildtype Creb2 to activate transcription.

We used three distinct assays to demonstrate that IPC-specific PKA pathway inhibition during larval development increases IIS. First, we measured larval weight gain, which is increased by IIS, by weighing developmentally-staged larvae at specific times after egg laying (AEL). We found that dilp2>PKAR* and *dilp2>Creb2^DN^* larvae grew faster than wildtype controls (Figure 1B). For example, by 108 hours AEL, dilp2>PKAR* and *dilp2>Creb2^DN^* were about 40% heavier than *dilp2>YFP*. Second, we used quantitative RT-PCR (Q-PCR) on developmentally-staged larvae to measure transcript levels of the *Thor* gene, which encodes initiation factor 4E binding protein and is repressed transcriptionally by IIS [15]. We found that *Thor* transcript levels were decreased in dilp2>PKAR* and *dilp2>Creb2^DN^* compared to *dilp2>YFP* 108 and 120 hours AEL (Figure 1C). Third, we used Western blot analysis levels on developmentally staged larvae to measure amount of the phosphorylated form of the kinase Akt (p-Akt), which is increased by IIS-mediated PI3 Kinase (PI3K) activation [16]. We found that p-Akt levels were substantially increased in dilp2>PKAR* and *dilp2>Creb2^DN^* larvae compared to *dilp2>YFP* 108 and 120 hours AEL (Figure 1D and not shown). Thus we conclude that blocking PKA pathway activity in the IPCs increases IIS.

Despite the more rapid growth in dilp2>PKAR* and *dilp2> Creb2^DN^* larvae, final pupal and adult size was altered only modestly. The dilp2>PKAR* and *dilp2>Creb2^DN^* genotype did not significantly affect either female or male adult weight (Figure 1E and data not shown). However, the dilp2>PKAR* and *dilp2> Creb2^DN^* female and male pupae were about 7% shorter than wildtype (Figure 1F and data not shown), and the resulting decreased length to weight ratio in many cases gave dilp2>PKAR* and *dilp2>Creb2^DN^* pupae a “chubby” appearance (Figure 1G).

### Increased IIS advances the onset of metamorphosis by precocious activation of ecdysone synthesis

Why did the increased growth rate in dilp2>PKAR* and *dilp2>Creb2^DN^* larvae fail to increase pupal or adult size? It was previously shown that activation of PI3K specifically within the prothoracic gland (PG) caused a precocious metamorphosis [4], [6] as a consequence of increased transcription of at least two ecdysone biosynthetic genes, *disembodied (dib)* and *phantom (phm)* [5] and hence precocious ecdysone synthesis. By attenuating the duration of the larval stages, this precocious metamorphosis decreased the amount of time available for larval growth and was at least partly responsible for the resulting dwarf phenotype.

**Figure 2:**
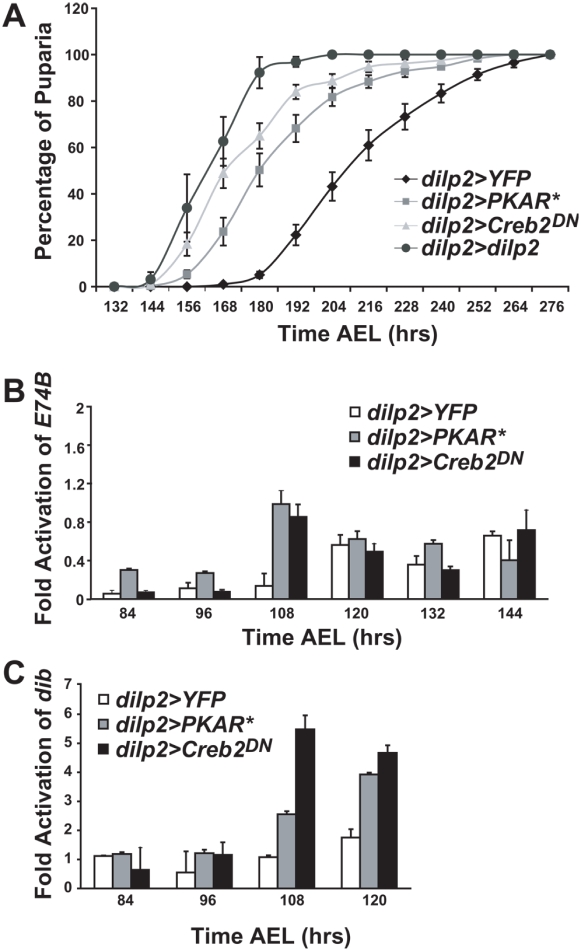
Increasing IIS causes precocious metamorphosis. (A) Mean fraction of developmentally staged larvae pupariating (y-axis) by the indicated time after egg laying (AEL, x-axis). Error bars represent SEMs, genotypes are listed in the inset. At least six independent biological samples were measured for each data point. (B,C) Mean *E74B* and *dib* transcript levels (y-axis), respectively, were measured from two biological samples collected from developmentally staged larvae of the indicated genotype (x-axis) and measured in triplicate at the indicated time AEL. Error bars represent SEMs. Data were obtained by quantitative RT-PCR with the relative 2^−ΔΔCt^ method and normalized to *RpL13A*.

Given that PI3K is the major intracellular effector of IIS, we asked if increasing IIS might similarly cause a precocious metamorphosis via increased transcription of *dib* and hence precocious synthesis of ecdysone. We allowed developmentally staged larvae to proceed to pupariation and found that dilp2>PKAR* and *dilp2>Creb2^DN^* larvae pupariated and ultimately eclosed as adults about 36 hours before *dilp2>YFP* (Figure 2A and not shown). However, the duration of the pupal stage did not appear to be significantly affected by the PKAR* and *Creb2^DN^* transgenes. We suggest that the 36 hour attenuation of the larval growing stages prevented dilp2>PKAR* and *dilp2>Creb2^DN^* from increasing pupal or adult size despite the increased growth rate.

To determine if this precocious metamorphosis resulted from increased transcription of ecdysone biosynthetic genes and precocious ecdysone synthesis, we used Q-PCR on RNA prepared from developmentally staged larvae and found that dilp2>PKAR* and *dilp2>Creb2^DN^* larvae exhibited greatly increased transcript levels of *dib* (Figure 2C), and induced transcription of the ecdysone-inducible reporter gene *E74B* at least 12 hours before *dilp2>YFP* (Figure 2B). These results suggest that increased IIS advances the onset of metamorphosis by activating PI3K in the PG, thus potentiating transcription of *dib* and presumably other ecdysone biosynthetic genes, and causing precocious ecdysone synthesis.

The results described above demonstrate that PKA pathway inhibition in the IPCs both increases IIS and advances the onset of metamorphosis. To demonstrate that metamorphosis is advanced as a result of the increased IIS, and not an effect of PKA pathway inhibition distinct from increased IIS, we overexpressed *dilp2* in the IPCs by driving *UAS-dilp2* with *dilp2-Gal4*. This manipulation was previously demonstrated to increase IIS [18]. We found that *dilp2>dilp2* larvae exhibited both an increased growth rate (Figure 1B) and a precocious metamorphosis (Figure 2A) similar to dilp2>PKAR* and *dilp2>Creb2^DN^*. Furthermore, adult weight and pupal length were not significantly different from wildtype in *dilp2>dilp2* (Figures 1E and 1F). These observations confirm that increased IIS advances the onset of metamorphosis.

It was previously reported that increased IIS, caused by transgene-induced overexpression of dilps, increased body size [13], [17]. In particular, ubiquitous overexpression of *dilp*s, accomplished by the *arm-Gal4* driver, increased adult weight by up to about 50% [13]. We found that we were likewise able to increase adult weight by ubiquitous, *arm-Gal4*-dependent *dilp2* overexpression: male and female *arm>dilp2* adults (n≥7) weighed 28% and 30% greater, respectively than *arm>YFP* adults (not shown). Thus, it appears that ubiquitous *dilp* overexpression and IPC-specific *dilp* overexpression affect body size differently.

It was previously reported that *dilp2>dilp2* adults were about 25% longer than wildtype [17]. In contrast, we found no significant differences in length between *dilp2>dilp2* pupae and controls (Figures 1E and 1F). The discrepancy between our results and those previously reported might involve differences in composition of the rearing media: it was previously reported that responsiveness of flies to altered IIS appears to be exquisitely sensitive to rearing media [18]. To test this possibility, we grew larvae on media containing a high yeast concentration (35 g/L) and low carbohydrate concentration and at 25°C, similar to the conditions employed in [17] (Kweon Yu, personal communication). We found that under these rearing conditions, *dilp2>dilp2* and dilp2>PKAR* adult females were about 15% and 5%, heavier, respectively than *dilp2>YFP* (not shown). Furthermore, the advancement of metamorphosis reported in Figure 2A was maintained under these conditions. Thus, the effects of increased IIS on final body size are affected by the yeast/carbohydrate ratios in rearing media. However, even under these new growth conditions, we still do not observe final body size effects comparable to what was reported previously [17], suggesting that additional genetic or environmental variables affecting the responsiveness of larvae to increased IIS remain to be identified.

## Discussion

### Increased IIS advances the onset of metamorphosis

Our results lead to three conclusions. First, IIS is a rate-limiting step for metamorphosis. It was previously shown that decreased IIS delays metamorphosis [2], whereas here we show that increased IIS is sufficient to advance metamorphosis even in the absence of direct changes to other hormone systems. Second, it was previously shown that transgene-induced PI3K activation in the prothoracic gland (PG) advances metamorphosis by advancing the timing of ecdysone synthesis via transcriptional activation of ecdysone biosynthetic genes [4]–[6]. Here we show that increasing IIS by transgene manipulation in the insulin producing cells confers a similar advancement of metamorphosis by a similar mechanism. Third, under conditions of extremely high growth rates, we show that Drosophila larvae proceed through development rapidly, rather than form large pupae and adults.

### A second pathway, mediated by prothoracicotropic hormone (PTTH), also regulates the timing of metamorphosis

**Figure 3:**
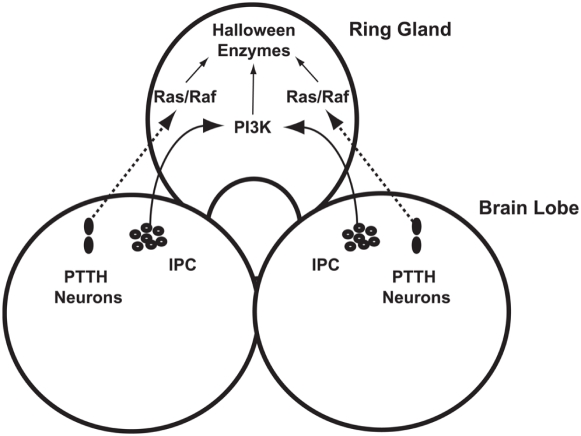
The regulation of ecdysone synthesis by insulin and PTTH. Brain lobes and the ring gland are indicated. Ecdysone synthesis is triggered when insulin released from the IPCs and PTTH released from the PG-LPs activate the PI3K and Ras pathways, respectively, in the PG. These two pathways, acting together, activate transcription of the PG-specific “Halloween genes”, which encode the ecdysone biosynthetic enzymes, ultimately triggering ecdysone synthesis. Arrows indicate activation pathways for which there is experimental evidence in Drosophila, hatched arrow indicates a speculative activation pathway.

The proper timing of metamorphosis requires a second hormone-mediated signalling pathway in addition to insulin. Genetic ablation of the neurons expressing prothoracicotropic hormone (PTTH) delay pupariation and metamorphosis as a consequence of attenuation of ecdysone biosynthetic gene transcription [19]. It was suggested [19] that PTTH communicates circadian time to the PG, thus linking metamorphosis to the circadian clock. The pathways within the PG mediating the response to PTTH are not known. However, previous results demonstrating that the Ras/Raf pathway in the PG regulates the timing of metamorphosis [4], taken together with experiments from the tobacco hornworm *Manduca sexta* that show that PTTH application increases levels of phospho-Erk, a target of Ras/Raf, within the PG [20], raise the possibility that PTTH activates ecdysone biosynthetic gene expression via the Ras/Raf pathway. In this view, full induction of ecdysone biosynthetic gene expression, and hence ecdysone synthesis and ultimately metamorphosis, requires both attainment of a specific body size and arrival at the proper time in the circadian clock, as communicated by IIS and PTTH regulating PI3K and Ras/Raf, respectively (Figure 3).

### Regulation of IIS by the PKA pathway

Our work suggests that PKA/Creb2 activity in the IPCs inhibits IIS. Although the extracellular ligands regulating IPC PKA activity are unknown, possible regulators include adipokinetic hormone (AKH), which has functional similarity to glucagon [21], [22], and serotonin or another factor produced in serotonergic neurons, which regulates IIS in larvae [23]. The AKH receptor is coupled to G_α_s and activates adenylate cyclase and PKA [21], whereas certain serotonin receptors inhibit adenylate cyclase [24]. Because AKH signalling is increased by starvation [21], [22], it seems possible that Akh might inhibit growth under nutrient limitation by activating PKA within the IPCs and hence downregulating insulin signalling,

The mechanism by which PKA/Creb2 inhibition activates IIS remains unknown. In mammals, Creb carries out both transcriptional activation and feedback inhibition of transcription at the insulin promoter [25]. Creb is also required for transcription of the insulin receptor substrate (IRS)-2 [8]. If the Drosophila IRS-2 within the IPCs is similarly decreased by PKAR* or *Creb2^DN^*, then absence of IRS-2 could increase insulin signalling by blocking insulin responsiveness and thus the recently-described [18] IIS-dependent negative feedback. Alternatively, PKA/Creb2 might regulate IIS by regulating the transcription of additional factors that potentiate insulin signalling, such as acid labile subunit [26] or ASNA-1 [27]. Further experiments will be required to address these issues.

### Regulating the timing of developmental transitions by growth rate in mammals

Altered growth rate, achieved by altered nutrition or endocrine disruptions, can also affect the timing of puberty in mammals. For example, genetic disruption of mouse growth hormone signalling, which decreases insulin growth factor (IGF) synthesis, delays puberty [28]. Similarly, delayed puberty observed in individuals with certain chronic diseases (for example, cystic fibrosis) has been attributed at least in part to difficulties in nutrient absorption, leading to disruptions in IGF signalling [29]. The observation that improved nutrition and increased growth rate have advanced the onset of puberty in humans in the west raises the possibility that increased IIS might be sufficient accelerate developmental transitions in humans as well as flies.

## Materials and Methods

### Drosophila stocks

All fly stocks were maintained on standard cornmeal/agar media at 22°C. The *dilp2-Gal4* line was provided by Eric Rulifson (Philadelphia, Pennsylvania), UAS-PKAR* was provided by Dan Kalderon (New York, New York), *UAS-Creb2^DN^* lines was provided by John Kiger (Davis, California) and *UAS-dilp2* was provided by Gyunghee Lee (Knoxville, Tennessee). All other stocks were provided by the Drosophila Stock Center at Bloomington, Indiana.

### Immunohistochemistry

Third instar larvae were dissected in 1× PBS, fixed in 4% formaldehyde in 1XPBS-T (0.1% TritonX) for 15 minutes at room temperature and washed 3 times, 10 minutes each time, in 1XPBS-T. Primary rabbit anti- phospho-CREB (Cell Signaling Technology, Inc. #9198) was incubated at 1∶50 overnight at 4°C. After three 10 minute 1XPBS-T washes secondary goat anti-rabbit antibody (Jackson Immunologicals) was incubated at 1∶500 for 2 hrs at room temperature. The samples were washed as above and mounted in UltraCruz mounting media containing DAPI (Santa Cruz, sc-24941). Images were collected using a Zeiss confocal laser-scanning microscope (40× objective).

### Larval staging

Larvae for Western blots, weight and Q-PCR were pre-laid for three days, and then were staged by serial transfer of parent flies (10 mating pairs per vial) at 12 hour intervals.

### Western blots

Staged larvae were collected, frozen and incubated on ice for 15 minutes in lysis buffer (120 nM NaCl, 50 mM Tris, 2 mM EDTA, 15 mM Na_4_O_7_P_2_, 20 mM NaF, 1.5 µM pepstatin A, 2.3 µM leupeptin and 100 µM PMSF). Samples were centrifuged at 18000 g for 30 minutes at 4°C. Equal amounts of protein lysates were aliquoted, 2× sample buffer was added, and samples were boiled for 5 min. Samples were then resolved on 8% SDS-PAGE gel and transferred to nitrocellulose. The blots were blocked in 5% non-fat milk and incubated with rabbit anti-*Drosophila* pAkt antibody at 1∶1000 (Cell Signaling Technologies) and rabbit anti-Akt antibody at 4°C overnight. Anti-vinculin 1∶500 was used as a loading control (Santa Cruz). HRP-conjugated secondary antibodies were used and chemiluminescence was detected on film, and digitized.

### Quantitative RT–PCR

RNA extractions from staged larvae, cDNA preparations and transcript analysis by Q-PCR were performed with the TaqMan gene expression assay (Applied Biosystems) as described in Caldwell et al. (2005). The *E74B* forward and reverse primers and probe sequences were described previously (Caldwell et al., 2005). To measure *dib* transcript levels, we used the forward primer sequence 5′GCCCAAGCTCACCAGATTGA3′, the reverse primer sequence 5′TGCAGACGAGCTCCAAAGGT3′ and the probe sequence 5′TTTGGAATTAACCTGTTTGCT3′. To measure *Thor* transcript levels we used the RPII140 expression assay (Applied Biosystems) and RPII 140 was used as the house keeping gene.
